# Anisotropy of Unstably Stratified Near-Surface Turbulence

**DOI:** 10.1007/s10546-021-00634-0

**Published:** 2021-06-15

**Authors:** Ivana Stiperski, Marcelo Chamecki, Marc Calaf

**Affiliations:** 1 Department of Atmospheric and Cryospheric Sciences, University of Innsbruck, Innsbruck, Austria; 2 Department of Atmospheric and Oceanic Sciences, University of California Los Angeles, Los Angeles, USA; 3 Department of Mechanical Engineering, University of Utah, Salt Lake City, USA

**Keywords:** Complex terrain, Reduced turbulence-kinetic-energy budget, Similarity scaling, Surface layer, Turbulence time scales

## Abstract

Classic Monin–Obukov similarity scaling states that in a stationary, horizontally homogeneous flow, in the absence of subsidence, turbulence is dictated by the balance between shear production and buoyancy production/destruction, whose ratio is characterized by a single universal scaling parameter. An evident breakdown in scaling is observed though, through large scatter in traditional scaling relations for the horizontal velocity variances under unstable stratification, or more generally in complex flow conditions. This breakdown suggests the existence of processes other than local shear and buoyancy that modulate near-surface turbulence. Recent studies on the role of anisotropy in similarity scaling have shown that anisotropy, even if calculated locally, may encode the information about these missing processes. We therefore examine the possible processes that govern the degree of anisotropy in convective conditions. We first use the reduced turbulence-kinetic-energy budget to show that anisotropy in convective conditions cannot be uniquely described by a balance of buoyancy and shear production and dissipation, but that other terms in the budget play an important role. Subsequently, we identify a ratio of local time scales that acts as a proxy for the anisotropic state of convective turbulence. This ratio can be used to formulate a new non-dimensional group. Results show that building on this approach the role of anisotropy in scaling relations over complex terrain can be placed into a more generalized framework.

## Introduction

**Fig. 1:**
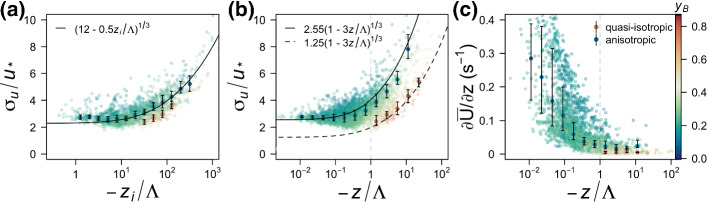
Flux–variance relation for standard deviation of the streamwise velocity component ($\sigma _u$) under **a** mixed-layer scaling with $z_i/{\Lambda }$ ($z_i$ is the mixed-layer height, and ${\Lambda }$ is the local Obukhov length) and **b** MOST with $z/{\Lambda }$. The scaling curves are obtained from Panofsky et al. (1977) and Kaimal and Finnigan (1994). **c** Mean vertical wind-speed gradient as a function of $z/{\Lambda }$. Data are from the T-Rex West tower, and are coloured according to $y_B$; $z_i$ is determined from the lidar measurements. Bin medians and inter-quartile ranges are calculated for quasi-isotropic conditions (orange) and anisotropic conditions (blue). For colour definition see Fig. 3

Strong thermal stratification presents a number of challenges to classic boundary-layer theories such as Monin–Obukhov similarity theory (MOST, Monin and Obukhov 1954), developed as a scaling framework for surface-layer turbulence. MOST rests on the hypothesis that a single length scale, the Obukhov length $L = - {u_*^3{\overline{T}}}/{\kappa \,g\overline{w'\theta _v'}}$ (Obukhov 1946) formed by the surface friction velocity ($u_* = ({\overline{u'w'}^2 + \overline{v'w'}^2})^{1/4}$), the surface buoyancy flux ($\overline{w'\theta _v'}$), and the buoyancy parameter $(g/\overline{\theta _v})$, can fully describe all the relevant processes within the surface layer. When combined with the height above the ground (*z*), *L* forms the sole surface-layer scaling parameter ${\zeta } = z/L$ (Monin and Obukhov 1954). As the most widely accepted scaling framework for surface-layer turbulence, MOST and its refinements and extensions (e.g., Businger et al. 1971; Kaimal et al. 1972; Kaimal 1973; Wyngaard and Coté 1971) have been extensively verified over the ideal, horizontally homogeneous, and flat terrain (i.e., canonical surface layer) for which they were developed (Kaimal and Wyngaard 1990). Early on, however, it was recognized that MOST suffers from a number of shortcomings. One of the most obvious failures is the lack of scaling of horizontal velocity variances (streamwise and spanwise) under unstable thermal stratification (e.g., Wyngaard and Coté 1974). Different reasons have been invoked to explain this failure. On the one hand, these include inherent differences in turbulence production by shear and buoyancy, where an upward energy cascade is hypothesized to lead to self-organization of convective turbulence into coherent structures (e.g., Zilitinkevich 1973; Zilitinkevich et al. 1998; Salesky et al. 2017; Zilitinkevich and Repina 2019). On the other hand, the failure has been attributed to neglected terms in the budgets of velocity variances such as effects of advection and dispersive fluxes due to thermal heterogeneities (e.g., Kröniger et al. 2019; Margairaz et al. 2020) or other non-local surface-related parameters (e.g., Wieringa 1986; Mason 1988; Claussen 1990; Avissar 1991; Beljaars and Holtslag 1991; Claussen 1991; Wood and Mason 1991; Avissar 1992; Blyth 1995; Bou-Zeid et al. 2004; Ament and Simmer 2006; Weigel et al. 2007). The most commonly accepted and widely used scaling approach for horizontal velocity variances, mixed-layer scaling, thus incorporates the mixed-layer height $z_i$ (e.g., Wyngaard and Coté 1974; Panofsky et al. 1977; Khanna and Brasseur 1997; Zilitinkevich et al. 2006; Banerjee et al. 2015; Chamecki et al. 2017) in recognition of the role of non-local large convective plumes spanning the depth of the convective boundary layer in determining the horizontal velocity variances. The mixed-layer approach indeed shows a significant reduction in scatter of data around the scaling curve (cf. Fig. 1a), compared with traditional MOST scaling (cf. Fig. 1b), but requires the knowledge of the mixed-layer height, which is both difficult to measure and, particularly in complex terrain, hard to define (Lehner and Rotach 2018). In addition, its usefulness in describing the variance of horizontal velocity components over complex terrain remains unresolved (Panofsky et al. 1977).

Using a local-scaling approach, where turbulence is scaled with values at the same measurement level, Stiperski and Calaf (2018) and Stiperski et al. (2019) recently showed that scaled horizontal velocity variances can in fact be reconciled with MOST if the information on the anisotropy of the Reynolds stress tensor is accounted for. Their results showed that one possible explanation for the commonly observed large scatter in scaling of horizontal velocity variances is the mixing of different anisotropic states, given that quasi-isotropic (three-component) turbulence scales with traditional MOST relations for the surface-normal variance ($\sigma _w$), while anisotropic two-component turbulence scales with the relation for the streamwise variance ($\sigma _u$) (cf., Fig. 1b). These results were shown to hold not only in the canonical surface layer but also in complex terrain (Stiperski et al. 2019), such as heterogeneous landscapes and mountainous topography, where experimental studies have consistently shown a breakdown of traditional MOST (e.g., Nadeau et al. 2013; Babić et al. 2016b, a; Rotach et al. 2017; Sfyri et al. 2018). Stiperski et al. (2019) demonstrated that, if clustered according to anisotropy, datasets representative of diverse flow and topographic forcing conditions that traditionally would not follow MOST scaling, fit the same scaling curve regardless of the complexity of the terrain or background forcing and without a need for the addition of a non-local scale. The degree of anisotropy was shown to explain up to $80\%$ of the departures of each dataset from the standard scaling curve, as indicated in Fig. 1b for the Terrain-induced Roto Experiment (T-Rex) dataset (Grubišič et al. 2008) where the distance from the standard scaling curve for the streamwise velocity component is clearly linked to the degree of anisotropy (colour). In fact, the mixed-layer scaling approach (Fig. 1a) also shows a clear stratification of data according to anisotropy, therefore highlighting its importance under any scaling framework. Anisotropy can explain multiple failures of MOST and allows traditional MOST scaling relations to embrace complexity.

How to unequivocally connect anisotropy to large-scale governing parameters, which would allow predicting the type of anisotropy that occurs in a set of given conditions, however, remains an open question. Stiperski and Calaf (2018) have shown that, for a canonical surface layer under unstable stratification, the anisotropy type is linked to terms of the turbulence kinetic energy (TKE) budget. They have shown that turbulence is quasi-isotropic in a regime dominated by buoyancy production ($-z/{\Lambda } > 1$), while two-component axisymmetric turbulence is found in near-neutral conditions with dominant shear production ($-z/{\Lambda } < 1$). Here, ${\Lambda }$ is the local Obukhov length (Nieuwstadt 1984a, b). Since momentum is transported by the anisotropic part of the Reynolds stress tensor (Pope 2000) this tendency for turbulence to become more isotropic with increasing instability is in line with the observed decrease in the efficiency of momentum transport (cf., Li and Bou-Zeid 2011) accompanied by a transition from convective rolls to convective cells (Salesky et al. 2017). In the canonical surface layer, the quasi-isotropic and anisotropic regimes do not overlap (Stiperski and Calaf 2018) and it is possible to identify a critical value of thermal stratification ($-z/{\Lambda } = 1$) for which this regime-transition occurs. This value has theoretical justifications as it falls within the range identified by Kader and Yaglom (1991) as transitioning between dynamic-convective and free convective sublayers. Furthermore, it corresponds to the onset of a regime in which the vertical velocity variance is the sole direct recipient of energy from the large-scale forcing, while horizontal velocity variances receive energy only through return-to-isotropy terms (Bou-Zeid et al. 2018). On the other hand, the results of Stiperski et al. (2019) hint that in complex terrain the stability parameter $z/{\Lambda }$ alone does not provide enough information to discern the conditions leading to two-component or quasi-isotropic turbulence. Even though quasi-isotropic turbulence still occurs only in highly unstable conditions ($-z/{\Lambda } > 1$, cf. Fig. 1), two-component turbulence is also found under equivalent thermal stratification. We can conclude that over non-canonical surfaces the degree of anisotropy is therefore no longer only controlled by the local production terms in the TKE budget (i.e., shear and buoyancy). Instead, processes described by additional budget terms must contribute significantly to maintaining turbulence anisotropy.

The study of these budget terms can be facilitated by a phase-space representation of the reduced TKE budget equation as recently introduced by Chamecki et al. (2018). This representation, in addition to shear production (*S*) and buoyancy production/destruction (*B*) terms, explicitly addresses the role of dissipation of TKE (${\varepsilon }$). Based on the hypothesis that the ratio between local shear production and TKE dissipation plays a critical role in scaling turbulence structure (Davidson and Krogstad 2014; De Silva et al. 2015; Chamecki et al. 2017), this approach goes beyond MOST in which one non-dimensional parameter is constructed from *S* and *B*, to provide two non-dimensional terms $S/{\varepsilon }$ and $B/{\varepsilon }$. Without explicitly assuming an equilibrium between production and dissipation of TKE, these two non-dimensional terms provide room for a potential generalization of surface-layer similarity theory and lay out a wider canvas for studying turbulence scaling under more generalized flow conditions.

The goal of this work is to study unstably stratified near-surface turbulence over complex terrain, and to identify key TKE budget terms and corresponding time scales that control different anisotropy regimes. These time scales and their non-dimensional ratio enable a revised and generalized version of traditional MOST, which offers an extension of scaling in disturbed flow conditions. For this purpose, we initially provide a brief overview of the reduced TKE phase space of Chamecki et al. (2018), as well as turbulence anisotropy classification as used in Stiperski and Calaf (2018) in Sect. 2. In Sect. 3, the datasets and post-processing methodology are presented, followed by the results in Sect. 4. Finally, we include a discussion of the results and their relevance with respect to traditional MOST scaling in Sect. 5, followed by the conclusions.

## A Brief Review of Some Key Theoretical Concepts

Here we provide a brief review of the key conceptual elements used in this work. For a complete description of the concepts, the reader is referred to the corresponding references.

### The Reduced Turbulence Kinetic Energy Phase Space

The reduced TKE budget (Chamecki et al. 2018) consists of a simplified representation of the TKE prognostic equation in which all the transport and transient terms are treated together as a residual (*R*) of the balance between local production and dissipation

1 $$\begin{aligned}&\underbrace{-\overline{u_{i}'u_{j}'}\frac{\partial {\overline{u}}_i}{\partial x_j}}_{S}+\underbrace{\frac{g}{{\overline{\theta }}_v}\overline{u_i'\theta _v'}\delta _{i3}}_{B}-{\varepsilon }\nonumber \\&\quad =\underbrace{\frac{\partial {\overline{e}}}{\partial t}+{\overline{u}}_i\frac{\partial {\overline{e}}}{\partial x_i}+\frac{\partial \overline{u_i'e}}{\partial x_i}+\frac{1}{\rho }\frac{\partial \overline{u_i'p'}}{\partial x_i}}_{R}. \end{aligned}$$

**Fig. 2:**
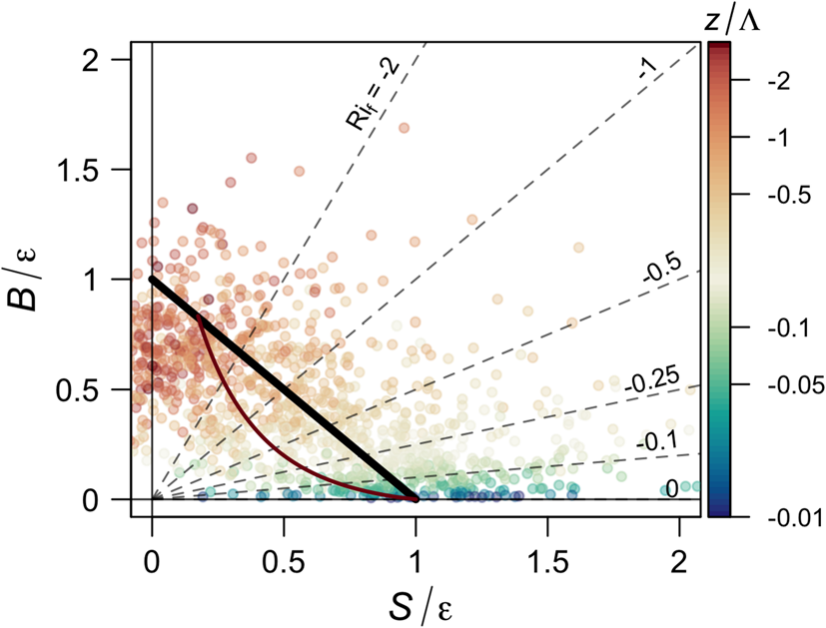
Reduced TKE phase space. The solid black line represents the states of local balance between production and dissipation and the dotted lines are lines of constant $Ri_f$. The points represent the data from the AHATS field campaign coloured according to the local stability parameter $z/{\Lambda }$. The thin brown line indicates the trajectory of the MOST function for non-dimensional wind shear based on empirical functions from the Kansas field campaign. Figure adapted from Chamecki et al. (2018). The small differences to the figure in Chamecki et al. (2018) are due to less strict quality criteria adopted here

Here, $e=(1/2)u_i'u_i'$ so that ${\overline{e}}$ is the TKE, $\theta _v$ is the virtual temperature, and $i = 1,2,3$ refer to the streamwise, spanwise, and vertical directions. The reduced TKE budget is then normalized by the local TKE dissipation rate $\varepsilon$, yielding a relation between three dimensionless quantities

2 $$\begin{aligned} (S/{\varepsilon })+(B/{\varepsilon })-1=(R/{\varepsilon }), \end{aligned}$$

which can be represented on a two-dimensional phase space where turbulence is characterized by the values of $S/{\varepsilon }$ and $B/{\varepsilon }$ (Fig. 2). In the phase space, lines emanating from the origin represent the constant flux Richardson number $Ri_f = -B/S$ (with the value of $Ri_f$ increasing clockwise), while the states corresponding to the local balance between production and dissipation ($R/{\varepsilon }=0$) fall on a straight diagonal line (thick black line in Fig. 2). All states above this line correspond to an excess in local production, implying negative net transport or an increase in TKE with time. Conversely, states below the line correspond to an excess in local dissipation, implying either a gain due to transport or a decay of turbulence with time. Note that, in the case that the TKE remains stationary, the residual term is solely representative of different transport processes (advection by the mean flow or secondary circulations, and transport by turbulence and pressure), and can be loosely referred to as a transport term.

In complex terrain where the flow is terrain-following, the TKE budget can be written in sloping coordinates (cf., Oldroyd et al. 2016) so that *u* is the streamwise and *w* the surface-normal velocity component. Under the assumptions of a uniform slope angle, the buoyancy term *B* in Eq. 1 needs to be adjusted to include the contribution from the streamwise heat flux $\overline{u'\theta _v'}$,

3 $$\begin{aligned} B = \frac{g}{{\overline{\theta }}_v}(\overline{w'\theta _v'}\cos \alpha - \overline{u'\theta _v'}\sin \alpha _{1}), \end{aligned}$$

where $\alpha$ is the slope angle, and $\alpha _{1} = \arcsin (\mathrm {cos}\psi \mathrm {sin}\alpha )$ incorporates the information on the deviation of the wind direction from the orientation of the slope. Here, we have neglected the contribution from $\overline{v'\theta _v'}$.

In the canonical surface layer where $R/{\varepsilon }$ is negligible, the data tend to scatter around the local balance line (see Advection Horizontal Array Turbulence Study, AHATS data in Fig. 2) and Eq. 2 indicates that turbulence can be fully characterized by one single dimensionless quantity ($S/{\varepsilon }$, $B/{\varepsilon }$, or their ratio $Ri_f$). In this case, the turbulence dissipation or memory time scale ${\mathcal {T}}_{{\varepsilon }}={\overline{e}}/{\varepsilon }$ is completely determined by the time scales associated with shear production (e.g., ${\mathcal {T}}_S\sim {\overline{e}}/S$) and buoyancy production/destruction (e.g., ${\mathcal {T}}_B\sim {\overline{e}}/B$), and MOST can be formally justified. A formal link to MOST can be made via specification of the similarity functions for shear and dissipation, which determine a line within the phase space, as illustrated in Fig. 2. Thus, MOST represents a reduction in the dimensionality of the phase space. In the general case in which the local imbalance ($R/{\varepsilon }$) is important, (at least) one additional time scale must be introduced to represent the dominant process causing the imbalance (${\mathcal {T}}_{R}$). For example, within the mixed-layer framework for horizontal velocity variances, this imbalance stems from turbulence and pressure transport by large convective eddies. Thus the distance from the balance line can be used as a measure of the importance of terms such as turbulent transport in closing the TKE budget. This approach can be useful when vertical turbulent transport plays a dominant role in the local TKE budget (Freire et al. 2019), but suffers if non-local terms have opposite signs and partially cancel out in the budget (Chamecki et al. 2020).

In more complex flows marked by complex topography or spatial variability of surface properties, mean advection is potentially the leading term in the local imbalance, and it may be more advantageous to adopt a Lagrangian interpretation of the TKE budget. In this view, ${\mathcal {T}}_{{\varepsilon }}$ represents the turbulence memory time scale, interpreted here as the time it takes for an eddy produced by the interaction with the mean flow to lose its identity through nonlinear interactions with its neighbour (Kaimal and Finnigan 1994). In dispersion modelling, this time scale serves as a proxy for the Lagrangian integral time scale (cf., Massman and Weil 1999).

The time scale ${\mathcal {T}}_{{\varepsilon }}$ can be contrasted to a time scale associated with the local imbalance. In the case where the imbalance is dominated by advection, the corresponding time scale is the advection time scale, defined as ${\mathcal {T}}_{A}=L_s/{\overline{u}}$, where $L_s$ is a horizontal distance characterizing changes in topography or surface properties, and ${\overline{u}}$ is the mean advective velocity. If ${\mathcal {T}}_{{\varepsilon }}/{\mathcal {T}}_{A}\ll 1$, energy dissipation is much faster than advection, so that turbulence quickly adapts to the local forcings and maintains an approximate state of local equilibrium. Conversely, if ${\mathcal {T}}_{{\varepsilon }}/{\mathcal {T}}_{A}\gg 1$, air parcels are transported through regions of changing forcing much faster than they can adjust to local conditions, thus turbulence has a long memory and the local TKE budget is strongly affected by upwind conditions.

In general, in flows where turbulent transport or advection is important, at least one additional time scale needs to be included, and two or more dimensionless quantities are needed to fully characterize turbulence energetics.

### Anisotropy and its Use for Data Classification

Turbulence anisotropy, determined through the six independent terms of the Reynolds stress tensor, provides a measure of the deformation of turbulent eddies with respect to their isotropic state (i.e., equal distribution of energy in all directions). This deformation and attendant anisotropy are responsible for effectively transporting momentum (Pope 2000).

Lumley and Newman (1977) have shown that, instead of using the six independent terms of the Reynolds stress tensor, anisotropy can be equivalently quantified through the two non-zero invariants of the anisotropy tensor, defined through its elements,

4 $$\begin{aligned} b_{ij} = \frac{\overline{u_i'u_j'}}{2{\overline{e}}} - \frac{1}{3}\delta _{ij}. \end{aligned}$$

Here, the anisotropic contribution of the Reynolds stress tensor is obtained by subtracting the isotropic part of the stress tensor and normalizing by twice the TKE.

**Fig. 3:**
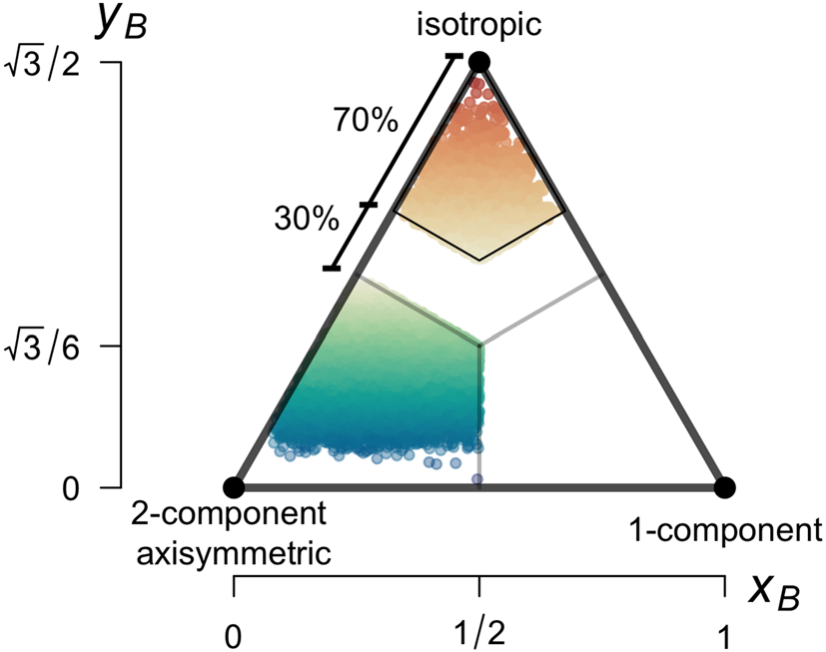
Barycentric representation of the Lumley triangle defined through the invariants $x_B$ and $y_B$ and its three limiting states of isotropic, two-component axisymmetric, and one-component turbulence. Points correspond to individual averaging periods coloured according to the value of $y_B$. We define data to be quasi-isotropic (warm colours) if falling within the kite defined by $70\%$ of half the side of the triangle from the isotropic vertex as in Stiperski and Calaf (2018). Anisotropic (i.e., two-component) data correspond to cooler colours. Data are from the METCRAX II experiment

Given that the anisotropy tensor is of rank two, it has three independent invariants that can be defined from its three eigenvalues. Due to its symmetric nature, however, the first invariant (the trace of the matrix) is identically zero. The two remaining invariants can be used to construct a two-dimensional phase space (anisotropy invariant map) that encompasses any realizable turbulence state (Pope 2000). The most commonly used and original anisotropy invariant map is the Lumley triangle (Lumley and Newman 1977). The nonlinear nature of the Lumley triangle, however, means that the limiting states are not given equal weight. We therefore use an equivalent set of invariants ($x_B$, $y_B$) that form a linear map that is the barycentric representation of the Lumley triangle (Banerjee et al. 2007). The barycentric invariants are defined as

5 $$\begin{aligned} x_B= & {} \lambda _{1} - \lambda _{2} + \frac{1}{2}(3\lambda _{3} + 1), \end{aligned}$$

6 $$\begin{aligned} y_B= & {} \frac{\sqrt{3}}{2}(3\lambda _{3} + 1), \end{aligned}$$

where $\lambda _1, \lambda _2, \lambda _3$ are the eigenvalues of the anisotropy tensor sorted in descending order. Any turbulent state can therefore be represented by a point with coordinates ($x_B,y_B$) on the barycentric map as in Fig. 3, while the vertices of the triangle define the three limiting states of anisotropy: isotropic ($x_B,y_B$) = ($1/2,\sqrt{3}/2$), two-component axisymmetric ($x_B,y_B$) = ($0,\,0$), and one-component turbulence ($x_B,y_B$) = ($1,\,0$). These limiting states are often graphically interpreted through a sphere (isotropic), line (one-component), and circle (two-component).

Following Stiperski and Calaf (2018) and Stiperski et al. (2019), we classify turbulence events as quasi-isotropic if turbulence anisotropy is within a distance of $70\%$ of half the side of the equilateral triangle from the isotropic vertex (warm colours in Fig. 3), and anisotropic if the turbulence state falls between the two-component axisymmetric limit and the centre of the barycentric map (cold colours in Fig. 3).

## Data and Methods

Datasets used originate from six multi-level towers in five well-known measurement campaigns with varying degree of terrain complexity. These cover the canonical flat and horizontally homogeneous terrain, as well as gently sloping terrain and floors of mountain valleys of different sizes. The datasets include AHATS (Salesky and Chamecki 2012), the Cooperative Atmosphere − Surface Exchange Study 1999 (CASES-99; Poulos et al. 2002), the Second Meteor Crater Experiment (METCRAX II; Lehner et al. 2016), T-Rex (Grubišič et al. 2008), and the Innsbruck Box (i-Box; Rotach et al. 2017). A detailed description of the datasets is given in Table 1 and a more in-depth discussion of individual characteristics can be found in Stiperski et al. (2019).

**Table 1:** Information on the datasets used

Station name	Official name	Terrain	Measurement heights [m]	Slope angle [$^{\circ }$]	Surface type	Data length
AHATS	AHATS	Flat	1.55, 3.3, 4.24, 5.53, 7.08, 8.05	0	Fallow land	June–August 2008
CASES99	CASES-99	Flat	5, 10, 20, 30, 40, 50, 55	0	Grassland	October 1999
T-RexC	Central Tower	Valley floor	5, 10, 15, 20, 25, 30	0.2	Desert	March–May 2006
i-Box0	CS-VF0	Valley floor	4, 8.7, 16.9	0.1	Mixed agricultural	January–December 2015
METCRAX	NEAR Tower	Slope	5, 10, 15, 20, 25, 30, 35, 40, 45, 50	1	Desert	October 2013
T-RexW	West Tower	Slope	5, 10, 15, 20, 25, 30	3.25	Desert	March–May 2006

All datasets were analyzed using the same processing procedures (cf. Stiperski et al. 2019) to ensure comparability. These include: a 30-min averaging period and detrending the data prior to block averaging. In addition, the coordinate system was rotated into the mean wind direction using double rotation for the i-Box and CASES99 datasets, while for the tilt corrected datasets (AHATS, METCRAX, T-Rex), only one rotation was used to rotate the data into the mean wind direction. No flux corrections were applied to the data.

While *S* in Eq. 1 includes the total (vertical and horizontal) shear production $S = S_v + S_h$, in keeping with the turbulence studies in canonical conditions, we use *S* to signify vertical shear production only, viz. $S \approx S_v = -\overline{u'w'}{\partial {\overline{U}}}/{\partial z}$, where ${\overline{U}}$ is the mean wind speed. The horizontal shear terms $S_h = S - S_v$ are thus assumed to be part of the residual term *R*. The vertical gradient of the wind speed needed for *S* at each measurement height was determined by fitting an analytical function $u(z) = a + b\,\ln {z} + c\,(\ln {z})^2$ to wind speeds at each height and calculating the derivative analytically. This function sometimes showed larger departures from the measurements close to the surface, and in that case we used a log–linear fit $u(z) = a + b\,z + c\,\ln {z}$ for the lowest two levels together with the roughness length. The chosen datasets were required to have a minimum of three measurement levels at each tower to ensure that the analytical fitting functions were not over-determined.

The time scale corresponding to the peak in the spectrum of the surface-normal velocity component (defined in Sect. 4.3) was determined as the inverse of the frequency at which the function $fS_w(f) = a\,f/(1 + b\,f^{5/3})$, fit to the measured surface-normal Fourier spectrum, has a maximum.

The TKE dissipation rate $\varepsilon$ was calculated from the second-order structure function (Chamecki and Dias 2004) as this method produced more consistent results compared with the spectral method.

Data were quality controlled to include stationarity of the mean wind speed, momentum flux and buoyancy flux using the stationarity test of Foken and Wichura (1996) as in Stiperski et al. (2019). Additionally, we exclude one-component turbulence (lower right vertex in Fig. 3) as this was shown to mostly belong to night-time unstable counter-gradient fluxes (cf., Stiperski and Calaf 2018). Averaging periods for which the slope of the second-order structure function in the inertial subrange deviated from two-thirds law by more than $10\%$ were also filtered out.

Finally, we look at data in a local sense (Nieuwstadt 1984a, b) by non-dimensionalizing variables with fluxes at the same measurement level, as already illustrated by the use of the local Obukhov length.

## Results

### Stability Parameter as a Necessary but not Sufficient Condition for Isotropy

As discussed in the introduction, while it is clear that quasi-isotropic turbulence occurs only for $z/{\Lambda } > 1$ when the vertical wind shear is negligible $\partial {\overline{U}}/\partial z \approx 0$ (Fig. 1c; cf. Stiperski et al. 2019), anisotropic turbulence coexists for the same conditions, although for marginally larger values of vertical wind shear. This result suggests that processes other than local shear and buoyancy (e.g., heterogeneity, different aspects of topography, etc.) cause turbulence to maintain anisotropy in highly convective conditions in complex terrain.

**Fig. 4:**
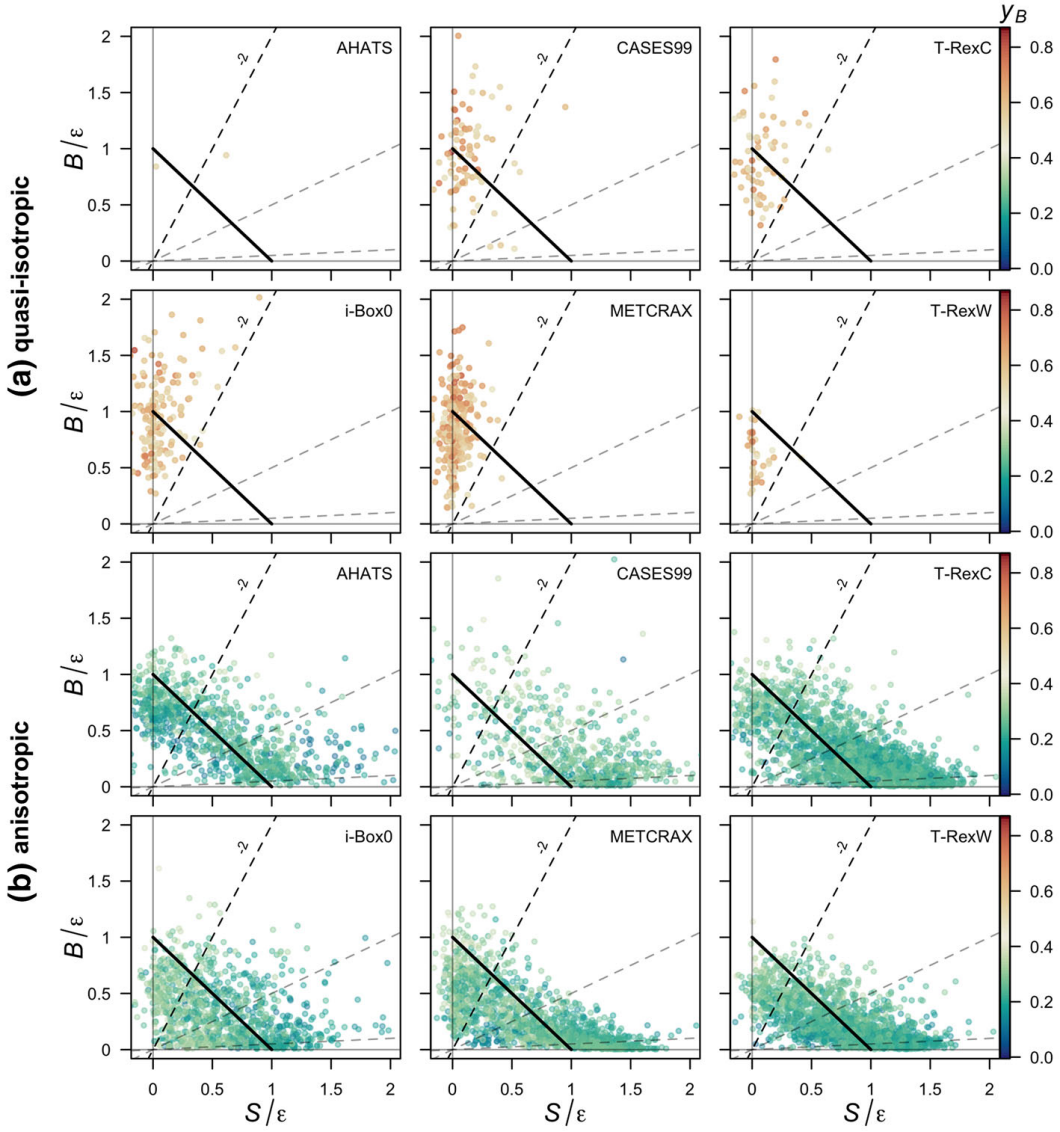
Reduced TKE space separated for quasi-isotropic **(a)** and anisotropic **(b)** data for all datasets, with data coloured as a function of anisotropy represented through $y_B$. The dashed lines indicate different Richardson numbers, from bottom to top, $Ri_f = [-0.05, -0.5, -2]$. The thick diagonal solid line indicates the region of balance between normalized shear production and buoyancy production/destruction

We therefore use the reduced TKE phase space (Chamecki et al. 2018) to investigate whether additional information from the TKE budget can help shed light on the differences in anisotropy types (Fig. 4). To facilitate visualization, we separate the data into quasi-isotropic (Fig. 4a) and anisotropic (Fig. 4b). In addition, we indicate the flux Richardson numbers relevant for transitions between the different energy-redistribution regimes identified by Bou-Zeid et al. (2018) $Ri_f = [ -0.05, -0.5, -2]$. Results show that quasi-isotropic turbulence is found in all the datasets irrespective of terrain and flow complexity, and occurs solely for $-Ri_f >\, 2$. According to Bou-Zeid et al. (2018), this is the limit beyond which buoyancy is the dominant production mechanism for TKE, and confirms the universality of results of Stiperski and Calaf (2018) who found isotropic turbulence only in buoyancy-driven conditions. The anisotropic data, on the other hand, are spread over a large portion of the phase space, and thus populate all the three regimes of Bou-Zeid et al. (2018). The results thus show no clear correlation between the degree of anisotropy (colour) and stability ($-Ri_f$), and confirm that stability alone is not governing anisotropy. The dominance of buoyancy production is therefore clearly a necessary, but not sufficient, condition for the existence of quasi-isotropy.

Hereafter, we divide the data into three groups: quasi-isotropic (with $$ -Ri_f >\, 2$$), convective anisotropic (data for which $$-Ri_f >\, 2$$ but not isotropic), and sheared anisotropic (defined as anisotropic turbulence in the presence of shear production; $$ 0<\,-Ri_f <\, 2$$). We explore the attributes of each group with the goal of explaining the existence of anisotropic turbulence in the absence of vertical shear production. Focusing on data with $$ -Ri_f >\, 2$$, a clear difference between quasi-isotropic (Fig. 4a) and convective anisotropic data (Fig. 4b above the line $$ -Ri_f = 2$$) is that while the former is more or less uniformly spread across the local balance line, the latter is predominantly below the line. This suggests an excess of local dissipation in convective anisotropic turbulence that must be balanced by net positive transport. Rough estimates of vertical turbulent transport show that it is not the single dominant contributor to the local imbalance (it accounts, on average, for about 25% of the imbalance), suggesting that mean advection of TKE or horizontal shear production (Goger et al. 2018) may be more important. This leads to the hypothesis that, under convective conditions, the advection of anisotropic turbulence from upwind regions with larger shear production or relevant horizontal shear may cause the observed behaviour in the phase diagram. Without a horizontal array of turbulence stations, we cannot directly address this hypothesis, but instead examine it through proxies.

**Fig. 5:**
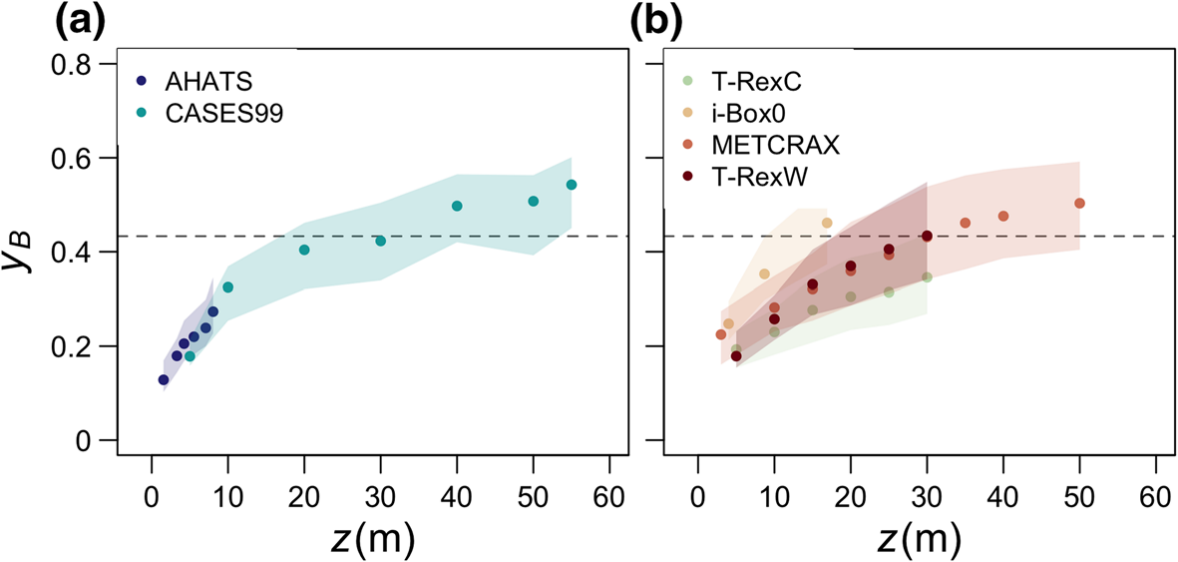
Dependence of anisotropy on height for $-Ri_f >\, 2$ for **a** canonical-surface-layer and **b** complex-terrain datasets. Points are medians and shading the interquartile range. The horizontal dashed line indicates the lowest reach of quasi-isotropy as defined in Fig. 3

Over flat and horizontally homogeneous terrain such as in the AHATS and CASES-99 experiments we can expect a negligible influence of advection by the mean flow. The cause of convective anisotropic turbulence there can be shown to be the closeness to the ground that results in turbulence deformation even under conditions of negligible shear. The distribution of anisotropy as a function of height under convective conditions $-Ri_f >\, 2$ (Fig. 5) therefore shows a clear trend of increasing isotropization of turbulence with increasing height, irrespective of the dataset. Although this trend is rather consistent, particularly in canonical conditions (Fig. 5a), the comparison of the degree of anisotropy between the datasets suggests that, for the same height range, anisotropy is more variable in complex terrain (Fig. 5b). In fact, the two T-Rex towers (T-RexC and T-RexW) that are only a couple of kilometres apart have strikingly different anisotropy for the same height. The interquartile ranges are also larger in complex terrain showing that turbulence there can be both quasi-isotropic and anisotropic for the same height range. We can therefore conclude that while height above ground is important in causing turbulence to be anisotropic very close to the surface, in complex terrain the non-local processes such as advection and horizontal shear maintain this anisotropy also at larger heights.

### Scalewise Characteristics of Anisotropy

**Fig. 6:**
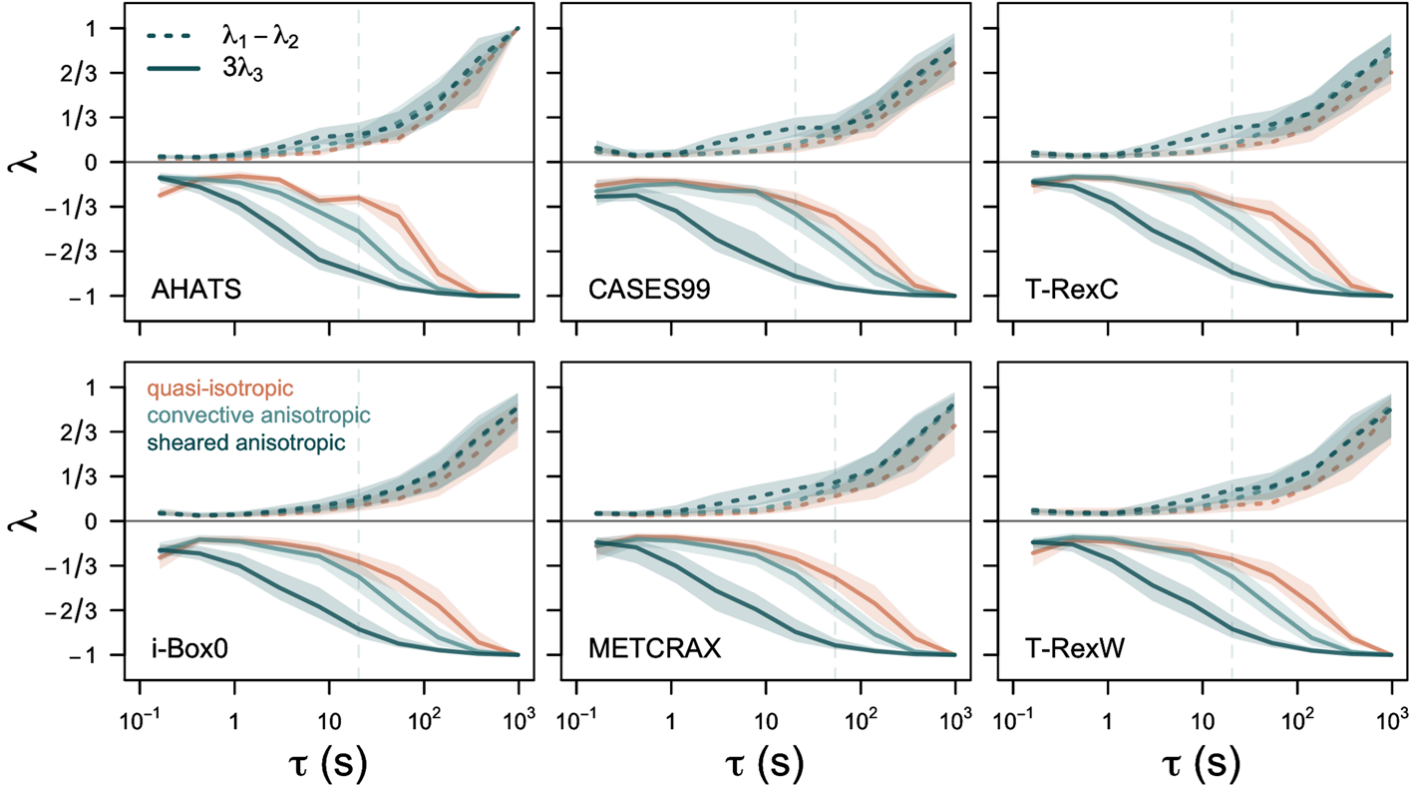
The difference between the two largest eigenvalues ($\lambda _1 - \lambda _2$, dashed line) and three times the smallest eigenvalue ($3\lambda _3$, full line) as a function of time scale $\tau$ obtained from the multi-resolution flux decomposition of the Reynolds stress. Colours correspond to quasi-isotropic, convective anisotropic, and sheared anisotropic turbulence. Vertical dashed line indicates the approximate maximum in the spectrum of the surface-normal velocity component *w* obtained from Fig. 7

**Fig. 7:**
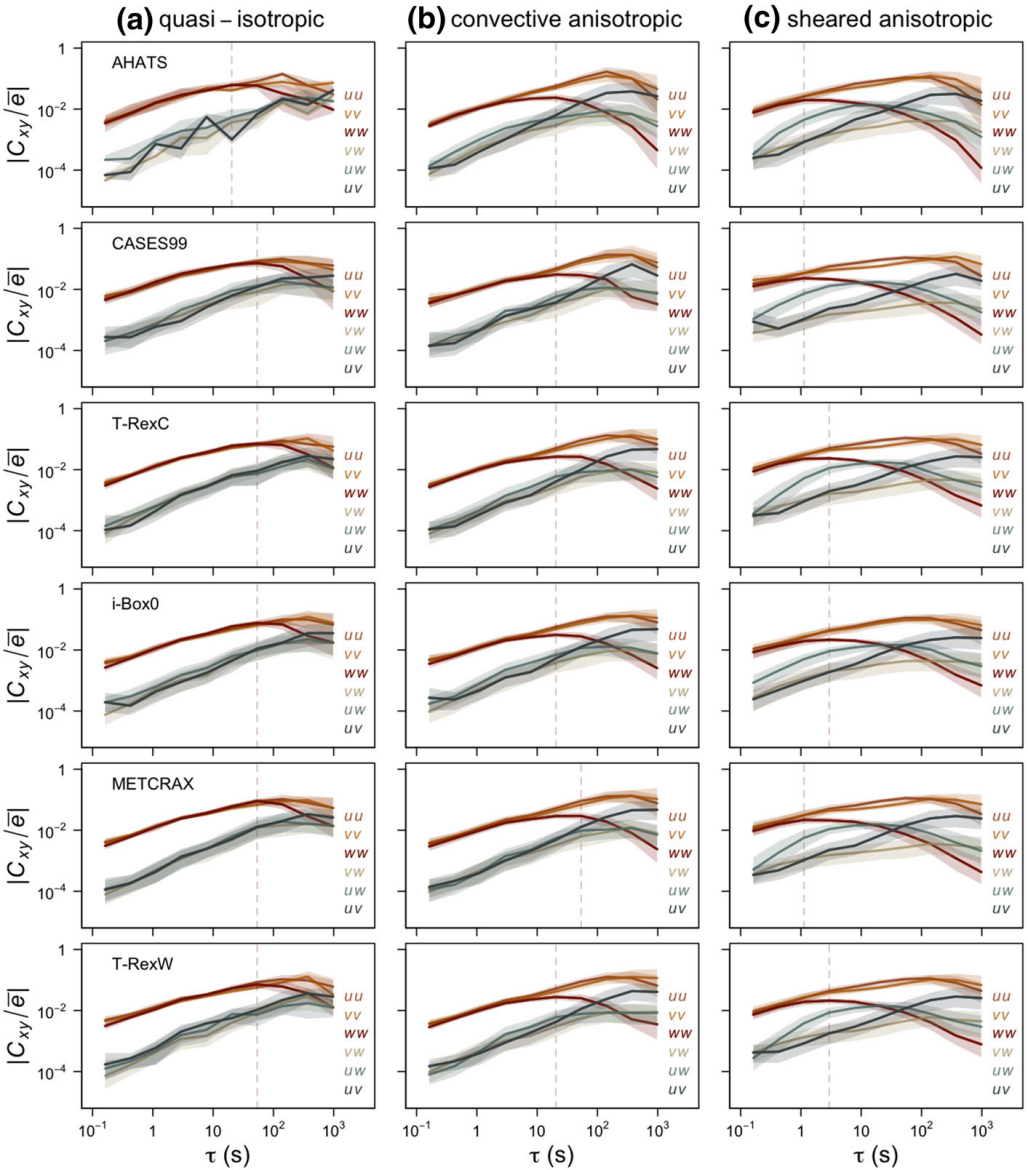
Cospectra of multi-resolution flux decomposition of different components of the Reynolds stress tensor $C_{xy}$ normalized by the TKE as a function of time scale $\tau$. For **a** quasi-isotropic, **b** convective anisotropic, and **c** sheared anisotropic turbulence. Vertical dashed line indicates the approximate maximum in the spectrum of the surface-normal velocity component *w*

In order to understand what causes the difference between the different isotropy types in the highly unstable regime, we next turn our attention to the scalewise distribution of energy between the different velocity components. We therefore compute the cospectra of the Reynolds stress tensor and eigenvalues of the anisotropy tensor using multi-resolution flux decomposition (Howell and Mahrt 1997; Klipp 2012, 2014; Stiperski et al. 2021) for different characteristic thermal stratification and types of anisotropy. Specifically, we are interested in identifying at which temporal scale ($\tau$) and for which components of the Reynolds stress tensor we can observe the differences between the quasi-isotropic, convective anisotropic, and sheared anisotropic turbulence (Figs. 6 and 7).

Figure 6 confirms that the largest turbulent scales examined here are anisotropic in all regimes, as already noted by Klipp (2012) and Klipp (2014), since both the value of $3\lambda _3$, which is proportional to anisotropy (cf., Eq. 6), and the difference between the two largest eigenvalues ($\lambda _1 - \lambda _2$) are close to one. For quasi-isotropic turbulence, however, the relaxation towards isotropy at decreasing scales is rapid and already at the time scale of approximately 20 s the eigenvalues approach a constant value in all the datasets (cf., Stiperski et al. 2021). Convective anisotropic turbulence exhibits a marginally less rapid approach to isotropy that starts at a somewhat smaller time scale, but plateaus around a similar time scale of approximately 10 s as isotropic turbulence. Thus, the results suggest that the source of anisotropy in convective conditions is constrained to the largest time scales. On the other hand, the smallest eigenvalue for shear anisotropic turbulence remains anisotropic until very small time scales ($< 1s$), while the difference between the two largest eigenvalues exhibits a bump in the mid-frequency range. This behaviour of eigenvalues appears to be universal among the datasets and only shows marginal differences as also observed by Stiperski et al. (2021).

To better understand the physical mechanisms behind the observed behaviour of eigenvalues, it is instructive to examine the scalewise contributions of different Reynolds stresses $C_{xy}$, where *x*, *y* represent different velocity components (Fig. 7). Although the individual Reynolds stresses are not invariant to the choice of the coordinate system, we can see a surprising degree of agreement between the datasets and thus extract a qualitative indication of the processes leading to anisotropy. Still, we highlight that our results are only representative of a streamline coordinate system in which the *w*-component is surface-normal.

Figure 7 shows that in quasi-isotropic turbulence ($-Ri_f > 2$) energy is equally distributed among the variances (i.e., $C_{uu}$, $C_{vv}$, $C_{ww}$) at all time scales, with only marginal differences at very large time scales ($\sim 10^2$ s, Fig. 7a), larger than those where the spectral peak is found. Similarly, the contribution of the covariance (off-diagonal) terms is equally weighted throughout all temporal scales, but with a significantly smaller magnitude than the diagonal terms, as expected for quasi-isotropy. The slight deviation from this picture for AHATS data is due to very few, and therefore potentially unrepresentative, isotropic data.

On the other hand, in the standard sheared anisotropic turbulence (Fig. 7c), the energy in the surface-normal velocity variance starts to deviate from the more energetic horizontal velocity variances already at very short time scales ($\sim \mathrm{l~s}$), in line with the time scale at which the smallest eigenvalue shows a decrease towards $-1$ (cf. Fig. 6). As expected for shear-driven turbulence, there is also a significant contribution of $C_{uw}$ at intermediate scales, coincident with the bump in $\lambda _1 - \lambda _2$, as well as a relevant increase of $C_{uv}$ at the largest scales.

Finally, the challenging case of convective anisotropic turbulence (Fig. 7b) lies in between the two limiting cases. While the streamwise and spanwise spectral contributions remain equal at all time scales, the spectra for the surface-normal velocity component have less energy at the larger scales than in the quasi-isotropic case, but significantly more than in the sheared anisotropic case, as already indicated by the eigenvalues and consistent with the blocking of eddies by the surface. In fact, the spectral maximum is located at time scales close to where the third eigenvalue diverges between isotropic and convective anisotropic turbulence (cf., Fig. 6). In addition, $C_{uv}$ shows the same behaviour as for sheared anisotropic turbulence, while the magnitude of $C_{uw}$ is only modestly higher than in quasi-isotropic turbulence. This results in a flow that behaves like two-component turbulence, although it should be quasi-isotropic according to thermal stratification. The reason seems to be that the existence of significant lateral shear through $C_{uv}$, as already observed by Klipp (2012), is leading the flow away from isotropy. Thus, despite the large Richardson number (and hence buoyancy), the flow is not dominated by a buoyancy time scale, but a significant contribution of the shear time scale remains. This shear time scale may originate from lateral shear production or from shear-generated turbulence that is advected from an upwind location, as suggested by the analysis of the reduced TKE phase space (cf. Sect. 2.1). From Fig. 7, it is also interesting to note that despite the datasets examined being representative of very different flow types and terrain complexities, the corresponding time scales at which the contribution of the surface-normal variance starts to deviate from the two horizontal variances are very similar, and are $\sim 10$ s. Clearly, the time scale associated with the peak in the spectrum of the surface-normal velocity component plays a key role here, as it distinguishes isotropic and anisotropic turbulence in highly convective conditions.

### Search for an Alternative Time Scale

In light of the results of the previous chapter (Figs. 4 and 7), and the corresponding theory presented in Sect. 2.1, the time scale associated with the peak in the spectrum of the surface-normal velocity component (herein referred to as ${\mathcal {T}}_{w_{max}}$) can be used to characterize the response of turbulence to both local processes (shear production and buoyancy production/destruction) as in the canonical-surface-layer case described in Sect. 2.1, and also non-local processes that form the imbalance term in the local TKE budget. This time scale is indicative of the largest turbulent eddies allowed to exist under the local thermal/shear, and non-local transport constraints. Since the surface-normal eddy size is constrained by the ground, this time scale is also a function of the height *z*.

At the same time, a second time scale exists and can be represented by the memory time scale (${\mathcal {T}}_{{\varepsilon }}$), which contains the information on the time it takes turbulence to attain an equilibrium state under the assumption of a constant strain rate (Kaimal and Finnigan 1994). Thus, for turbulence to be able to reach quasi-isotropy, not only is there a need for a dominance of local buoyancy over local shear such that $-Ri_f > 2$, but there should also be a certain relationship between the response time of turbulence (${\mathcal {T}}_{w_{max}}$) and the memory time scale (${\mathcal {T}}_{{\varepsilon }}$). When ${\mathcal {T}}_{{\varepsilon }}/{\mathcal {T}}_{w_{max}}<< 1$, turbulence memory is short and therefore turbulence is in equilibrium with the local surface allowing turbulence to reach quasi-isotropy far enough away from the wall. On the other hand, when ${\mathcal {T}}_{{\varepsilon }}/{\mathcal {T}}_{w_{max}}>> 1$, turbulence still remembers the upstream conditions and, therefore, we can expect anisotropy to be maintained even in highly convective conditions.

**Fig. 8:**
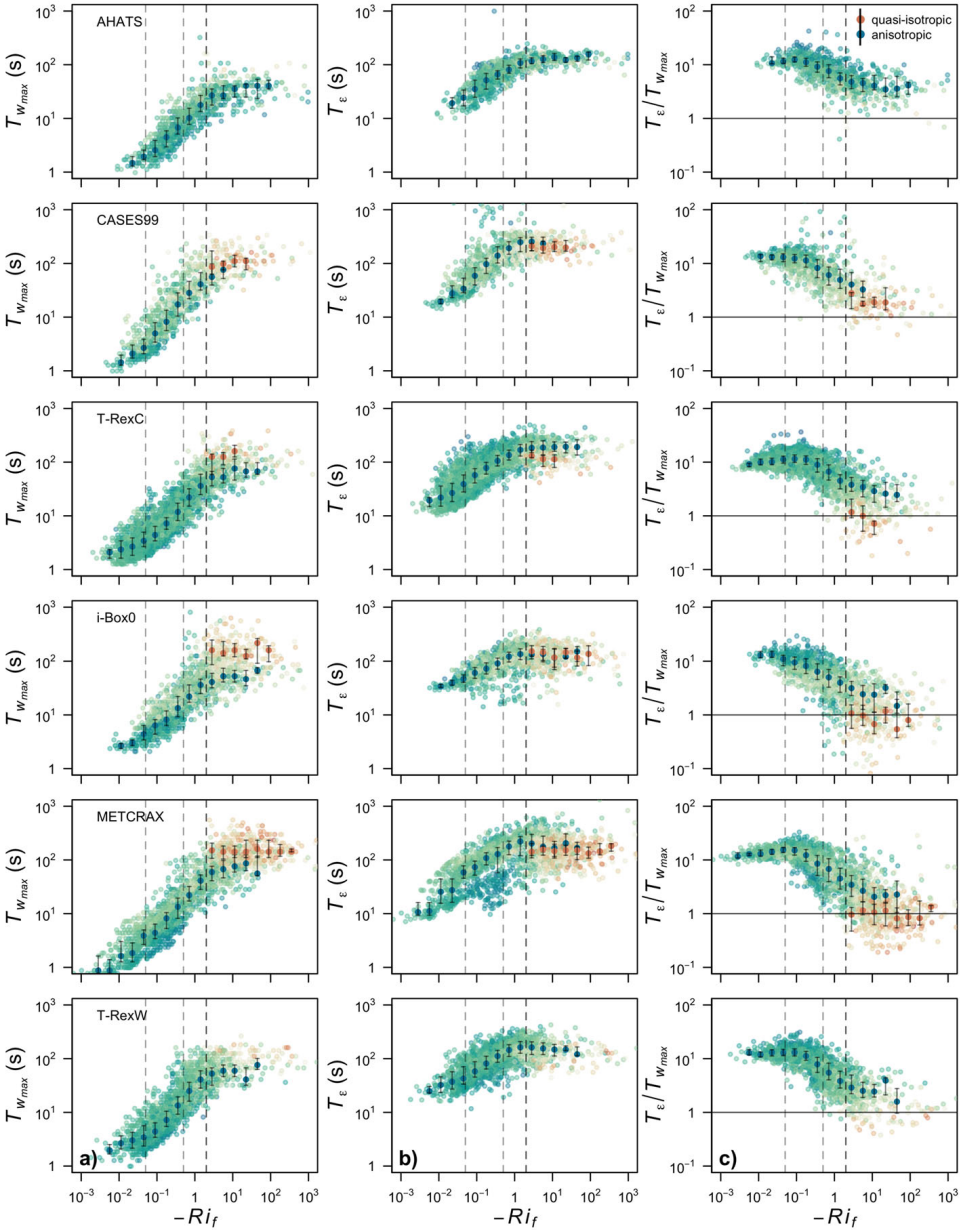
**a** Time scale of the most energetic eddies in the *w*-spectrum ${\mathcal {T}}_{w_{max}}$, **b** memory time scale ${\mathcal {T}}_{{\varepsilon }}$, and **c** their ratio as a function of $-Ri_f$ for all datasets. Colours of points correspond to values of $y_B$. Bin medians and inter-quantile ranges are shown for quasi-isotropic turbulence (orange) and anisotropic turbulence (blue). Vertical dashed lines indicate different thresholds of Richardson number, $-Ri_f = [ 0.05,0.5,2]$

We investigate this hypothesis in detail by examining the memory time scale (${\mathcal {T}}_{{\varepsilon }}$) and the response time scale (${\mathcal {T}}_{w_{max}}$) as a function of $Ri_f$ (Fig. 8). As one would expect, ${\mathcal {T}}_{w_{max}}$ progressively increases with increasing thermal stratification, from very low values in near-neutral stratification $-Ri_f < 0.05$, where $\overline{w'^2}$ is solely the recipient of energy from the streamwise component, to a saturation regime well above $-Ri_f = 2$, where it is the sole beneficiary of energy (Bou-Zeid et al. 2018). Given the correspondence of ${\mathcal {T}}_{w_{max}}$ with the integral time scale for the surface-normal variance, this saturation regime could be interpreted as the surface thermal forcing not having enough energy to maintain the progressive growth of convective eddies, and hence reaching a constant value. If one focuses only on quasi-isotropic data (warm colours in Fig. 8a), however, it is clear that the quasi-isotropic eddies always have longer time scales than those associated with anisotropic turbulence for the equivalent thermal stratification. A similar behaviour is observed for the memory time scale ${\mathcal {T}}_{{\varepsilon }}$ (Fig. 8b), which grows with increasing thermal stratification but at a much slower rate than ${\mathcal {T}}_{w_{max}}$ and starting from an initially significantly higher value. This time scale also reaches a saturation regime but already at $-Ri_f \approx 2$, after which it even starts to decrease slightly, as the loss of energy due to upscale cascade might become relevant (Zilitinkevich and Repina 2019). More importantly, one can also see that ${\mathcal {T}}_{{\varepsilon }}$ does not show any dependence on anisotropy. These characteristics of both time scales are observed in all datasets.

The corresponding ratio between the two time scales (${\mathcal {T}}_{{\varepsilon }}/{\mathcal {T}}_{w_{max}}$) is a clear function of anisotropy (Fig. 8c). It is only when the ratio is $\le 1$ that a quasi-isotropic state can be reached in all datasets, otherwise anisotropy is maintained. Furthermore, in most of the cases, this additional limit is only reached beyond $-Ri_f >2$. In fact, it is only on those more ideal sites, representative of idealized flow conditions such as CASES99, that ${\mathcal {T}}_{{\varepsilon }}/{\mathcal {T}}_{w_{max}} \le 1$ is reached simultaneously with $-Ri_f > 2$.

**Fig. 9:**
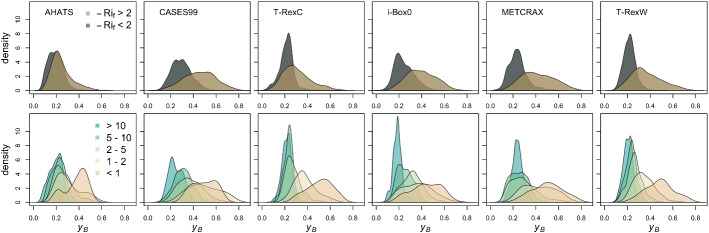
Probability density function of anisotropy ($y_{B}$) as a function of $-Ri_{f}$ (upper row), and ${\mathcal {T}}_{{\varepsilon }}/{\mathcal {T}}_{w_{max}}$ (lower row)

The fact that the ratio of time scales is a more relevant criterion for detecting the transition in anisotropy regimes than the Richardson number alone, is also shown in Fig. 9. Here the two probability distributions for anisotropy display significant overlap when classifying the data based on $Ri_{f}$ (for AHATS the two distributions are almost identical) but are clearly different when classifying data based on the ratio between time scales (${\mathcal {T}}_{{\varepsilon }}/{\mathcal {T}}_{{w}_{max}}$).

### New Non-Dimensional Group

**Fig. 10:**
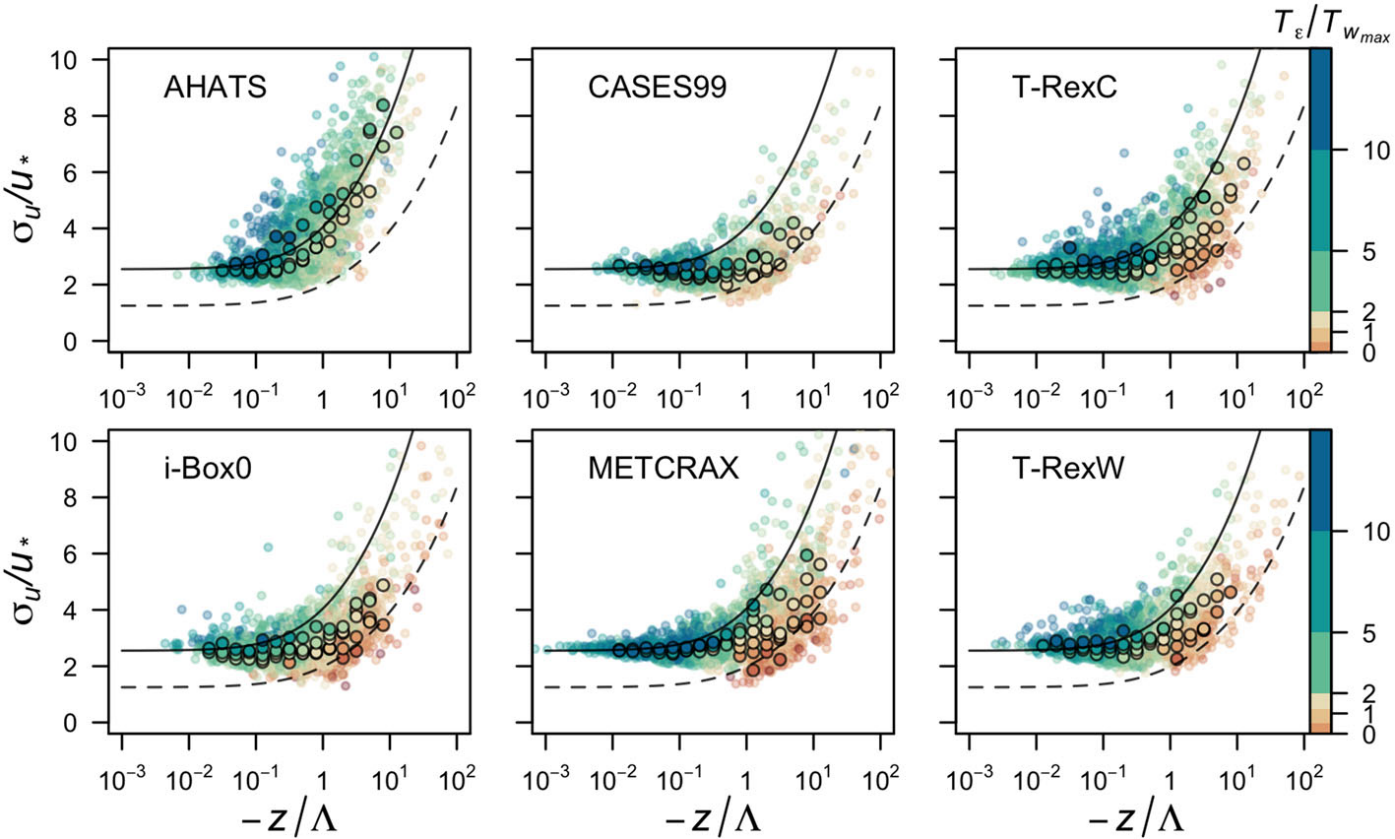
Flux–variance relation for $\sigma _u$ as a function of local stability ($z/{\Lambda }$) for all datasets. Data are coloured according to ${\mathcal {T}}_{{\varepsilon }}/{\mathcal {T}}_{w_{max}}$. Bin medians are represented by circles and are calculated for the categories [$\le 1$, $1-2$, $2-5$, $5-10$, $>10$] (cf. Fig. 9). The solid line corresponds to the $\sigma _u$ curve and dashed line to the $\sigma _w$ curve as in Fig. 1

The additional ratio of time scales ${\mathcal {T}}_{{\varepsilon }}/{\mathcal {T}}_{w_{max}}$ derived from the TKE equation can be used as a new non-dimensional group in scaling. We test its suitability on the scaling of the standard deviation of the streamwise velocity component by clustering the scaled data according to the ratio of time scales (Fig. 10). As we saw in Fig. 8, this ratio spans more than an order of magnitude and we therefore separate the data into five bins: $[\le 1, 1-2, 2-5, 5-10, >10]$. The results show that when the ratio is $\le 1$, data for $\sigma _u/u_*$ fit very well with the canonical curve for $\sigma _w$ (dashed line), equivalently to separating the runs based on $y_B$ (cf. Fig. 1). On the other hand, data for which the ratio $> 10$ fit better to the $\sigma _u$ line and correspond more closely to anisotropic turbulence. Thus we can confirm that the ratio of time scales contains relevant information on the processes that maintain anisotropy and can be used to detect its degree.

## Discussion

The results presented above have shown that maintenance of anisotropy under convective conditions, and therefore also relevant momentum transport, is a result of non-equilibrium of turbulence with its local surface forcings (shear and buoyancy). This results in a non-negligible contribution of $\overline{u'v'}$ in the Reynolds stress tensor and correspondingly a reduction in the surface normal velocity variance, which can be quantified with the ratio of time scales ${\mathcal {T}}_{{\varepsilon }}/{\mathcal {T}}_{w_{max}}$.

In this analysis, the time scale associated with the maximum in the surface-normal velocity spectrum (${\mathcal {T}}_{w_{max}}$) represents the response of turbulence to both the local terms in the reduced TKE budget (local shear and buoyancy production/destruction) as well as processes that form the residual or the imbalance. Based on the available data (single tower measurements), it is impossible to determine the specific sources of this imbalance, whether it is dominated by the turbulent transport, horizontal shear production, pressure correlation or the mean advection. The results presented in Figs. 4 and 7 suggest the importance of horizontal shear production and the advection of upstream shear. Still, additional spatially distributed data are needed to further disentangle the individual contributions to ${\mathcal {T}}_{w_{max}}$.

**Fig. 11:**
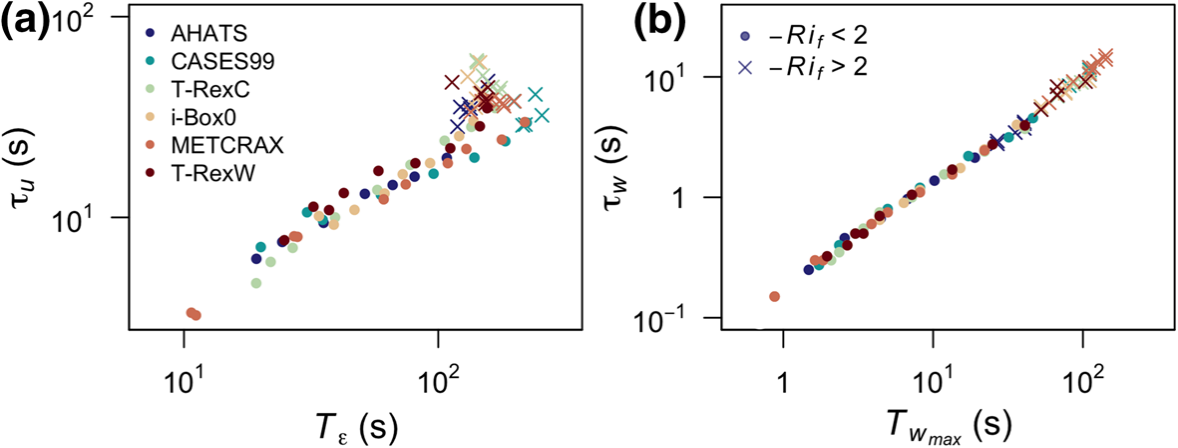
Relationship between **a** the streamwise Eulerian integral time scales $\tau _u$ with the corresponding memory time scale ${\mathcal {T}}_{{\varepsilon }}$, and **b** surface-normal Eulerian integral time scale $\tau _w$ and ${\mathcal {T}}_{w_{max}}$. The datasets are represented by colours. Points correspond to $-Ri_f<2$ and crosses to $-Ri_f > 2$

Finally, we can put our results within the context of Eulerian integral time scales in the streamwise ($\tau _u$) and surface-normal ($\tau _w$) directions (Fig. 11). As expected, ${\mathcal {T}}_{w_{max}}$ is almost perfectly correlated to $\tau _{w}$. However, ${\mathcal {T}}_{{\varepsilon }}$ used as a proxy for the Lagrangian integral time scale (cf. Hanna 1981) is only well-correlated with the streamwise Eulerian integral time scale ($\tau _{u}$) in the shear dominated region $-Ri_f<2$. The de-correlation of time scales in convective conditions shows that Taylor’s hypothesis is no longer valid and the Eulerian framework is no longer capturing the evolution of turbulence in quasi-isotropic conditions. The fact that the magnitude of $\tau _{u}$ continues to increase beyond $-Ri_f = 2$ where ${\mathcal {T}}_{{\varepsilon }}$ has a maximum, suggests that the redistribution of variance between the velocity components becomes faster and therefore more efficient for higher $Ri_f$ (Bou-Zeid et al. 2018). These results not only indicate that ${\mathcal {T}}_{{\varepsilon }}$ encodes different information than $\tau _{u}$ when $-Ri_f>2$, but also illustrate that the additional requirement of ${\mathcal {T}}_{{\varepsilon }}/{\mathcal {T}}_{w_{max}} \le 1$ developed in this work is non-trivial, and hence cannot be equated with the requirement that isotropy is expected when $\tau _{u}\sim \tau _{w}$.

## Conclusions

This work presents an extended analysis of the conditions required for turbulence to reach quasi-isotropy from a TKE budget perspective, with a specific focus on the implications for expanding MOST scaling into complex conditions, as hinted in Stiperski and Calaf (2018) and Stiperski et al. (2019). The results show that the scaling of horizontal velocity variances can be reconciled under MOST by an addition of the ratio of local scales related to anisotropy only. In a canonical surface layer, anisotropy is shown to be dependent on stratification, but, very close to the surface, turbulence remains anisotropic even in highly convective conditions. In complex terrain, however, it is not possible to establish a unique link between height, stratification, and anisotropy. Instead, the knowledge of the equilibrium of turbulence with its surface forcing is required. We can conclude that the initial requirement of $-Ri_f > 2$, while necessary, is not a sufficient condition for isotropy to occur, and the additional condition of ${\mathcal {T}}_{{\varepsilon }}/{\mathcal {T}}_{w_{max}} \le 1$ is needed. Through the combination of these two conditions, it is therefore possible to unequivocally determine when isotropy can be expected independently of complexity. This result will facilitate extending MOST scaling beyond traditional canonical conditions. Whilst at present the models using at least 1.5-order closure are able to parametrize only ${\mathcal {T}}_{{\varepsilon }}$, it can be expected that in the future it will be possible to also parametrize ${\mathcal {T}}_{w_{max}}$ (cf. Ayet and Katul 2020), thus allowing practical application of this research.
